# Oral perfluorooctane sulfonate (PFOS) lessens tumor development in the APC^min^ mouse model of spontaneous familial adenomatous polyposis

**DOI:** 10.1186/s12885-016-2861-5

**Published:** 2016-12-08

**Authors:** Jeffrey Wimsatt, Meghan Villers, Laurel Thomas, Stacey Kamarec, Caitlin Montgomery, Leo W. Y. Yeung, Yanqing Hu, Kim Innes

**Affiliations:** 1 Department of Medicine, School of Medicine, West Virginia University, Morgantown, WV 26506 USA; 2 Department of Epidemiology, School of Public Health, West Virginia University, Morgantown, WV 26506 USA; 3 Man-Technology-Environment (MTM) Research Centre, School of Science and Technology, Örebro University, Fakultetsgatan 1, Örebro, SE-70182 Sweden; 4 Department of Statistics, West Virginia University, Morgantown, WV 26506 USA; 5 West Virginia University, 186 HSCN, 1 Medical Center Drive, Morgantown, WV 26508 USA

**Keywords:** APC^min^ mouse, Perfluorooctane sulfonate, PFOS, Colorectal cancer, Dose–response, Gender

## Abstract

**Background:**

Colorectal cancer is the second most common cause of cancer deaths for both men and women, and the third most common cause of cancer in the U.S. Toxicity of current chemotherapeutic agents for colorectal cancer, and emergence of drug resistance underscore the need to develop new, potentially less toxic alternatives. Our recent cross-sectional study in a large Appalachian population, showed a strong, inverse, dose–response association of serum perfluorooctane sulfonate (PFOS) levels to prevalent colorectal cancer, suggesting PFOS may have therapeutic potential in the prevention and/or treatment of colorectal cancer. In these preliminary studies using a mouse model of familial colorectal cancer, the APC^min^ mouse, and exposures comparable to those reported in human populations, we assess the efficacy of PFOS for reducing tumor burden, and evaluate potential dose–response effects.

**Methods:**

At 5–6 weeks of age, APC^min^ mice were randomized to receive 0, 20, 250 mg PFOS/kg (females) or 0, 10, 50 and 200 mg PFOS/kg (males) via their drinking water. At 15 weeks of age, gastrointestinal tumors were counted and scored and blood PFOS levels measured.

**Results:**

PFOS exposure was associated with a significant, dose–response reduction in total tumor number in both male and female mice. This inverse dose–response effect of PFOS exposure was particularly pronounced for larger tumors (r^2^ for linear trend = 0.44 for males, *p’s* <0.001).

**Conclusions:**

The current study in a mouse model of familial adenomatous polyposis offers the first experimental evidence that chronic exposure to PFOS in drinking water can reduce formation of gastrointestinal tumors, and that these reductions are both significant and dose-dependent. If confirmed in further studies, these promising findings could lead to new therapeutic strategies for familial colorectal cancer, and suggest that PFOS testing in both preventive and therapeutic models for human colorectal cancer is warranted.

## Background

Colorectal cancer (colorectal cancer) is the second most common cause of cancer deaths in both men and women, and the third most common cause of cancer in the US [1]. Toxicity of current chemotherapeutic agents for colorectal cancer, and ongoing challenges with drug resistance suggest that new drug approaches continue to have value [2, 3].

Perfluoroalkyls and polyfluoroalkyls have been manufactured for over five decades; their unique-oil-repellence and high surface activity make them excellent surface protectants and surfactants. Some of these compounds are potent peroxisome proliferator-activated receptor (PPAR) ligands, and have demonstrated anti-inflammatory effects in vitro [4] and in animal studies [5]; these effects are thought to operate via both PPAR-dependent and independent pathways [6]. Perfluorooctane sulfonate, (PFOS, C_8_HF_17_O_3_S), a well-studied perfluoro-surfactant, is a widespread environmental contaminant, has been detected in the plasma of virtually every human population worldwide [7–9]. PFOS is extremely stable in the environment, readily accumulates in people and animals, and has toxic properties; as a result several countries have voluntarily joined the Stockholm Convention to stop its use [10]. Lifetime exposure studies in rodents suggest PFOS can cause liver adenomas, whereas the evidence for cancer induction in humans remains equivocal, perhaps in part because most exposures levels are so low [11]. Hence, if shown of benefit, particularly at low doses, PFOS could suggest a novel mechanism for treating colorectal cancer.

Recent research suggests that PFOS may also have value as a chemopreventive and/or chemotherapeutic agent for colorectal cancer. In a cross-sectional study in a large Ohio Valley cohort (the C8 Health Project), we investigated the potential link between prevalent colorectal cancer and serum PFOS [12]. PFOS levels in this population were similar to those reported in the general U.S. population [12, 13], were comparable to or lower than those reported from non-occupational settings in other countries (e.g. ≤ 30 ng/ml) [14, 15], and were well below levels reported in fluorochemical workers [16]. We found a strong, inverse, dose–response association between serum levels of PFOS and prevalent colorectal cancer that remained robust after adjustment for multiple possible confounders and persisted even at very low exposure levels [12]. However, while these findings suggest that PFOS may be protective against colorectal cancer, the cross-sectional nature of the data preclude determination of causality.

Here the potential chemotherapeutic value of PFOS is tested in APC^min^ mice, a genetic model for familial adenomatous polyposis) in humans [15, 16].

## Methods

### Chemicals and reagents

For animal studies, potassium salt of PFOS (Sigma #77282; heptadecafluorooctanesulfonic acid potassium salt) was purchased and dissolved in Millipore® water containing 0.5% Tween 20 (Sigma #P2287). Bottles were made fresh weekly by addition from a stock solution. For analytical purposes, perfluorohexanoate, perfluoroheptanoate, perfluorooctanoate, potassium salts of perfluorohexanesulfonate, perfluorooctanesulfonate, and ^13^C_4_ PFOS were obtained from Wellington Laboratories (Guelph, Ontario, Canada). The purity of all standards was over 98%. Tetrabutylammonium hydrogen sulfate (99%), ammonium acetate (>99%), and ammonia (NH_3_, 30%) were obtained from Sigma-Aldrich. LCMS grade methanol and methyl-tert-butyl ether (MTBE, > 99%) were acquired from EMD Chemicals Inc. (Mississauga, ON). Oasis® weak anion exchange (WAX; 6 cm^3^, 150 mg, 30 μm) solid phase extraction (SPE) cartridges were purchased from Waters (Milford, MA).

All studies were approved by the Institutional Animal Care and Use Committee at West Virginia University. Animals were housed individually in standard ventilated barrier caging and fed standard mouse chow, and maintained on a 12 : 12 (L : D) hour light cycle. In two separate studies, female and male APC^min^ mice (C57BL/6 J-Apc^Min^/J) were acquired from JAX at 6 and 5 weeks respectively, acclimated for 1 week, and randomized by treatment group. Animals in each group received Tween-20 vehicle or PFOS dissolved in Tween-20 in their drinking water. In an initial pilot study, female mice (*n* = 8/group) were exposed to 0.5% Tween-20 vehicle or Tween-20 with 20, or 250 mg/kg PFOS target doses in their drinking water from 7–15 weeks of age based on estimated daily water consumption. Similarly, in the second study, male mice were exposed to vehicle or PFOS target doses of 10, 50 and 200 mg/kg (all groups, *n* = 6) provided at 6–15 weeks of age. Animals were weighed twice weekly throughout the study period.

In both studies, animals were humanely euthanized with CO_2_, and the complete gastrointestinal tract from the stomach to the rectum was opened lengthwise and tumors were counted and categorized using direct visualization under 3 times magnification. Tumors were recognized by their characteristic gross morphology and categorized by location (small intestine, large intestine, cecum), size (using the average surface dimensions, they were scored as < 1 mm or ≥ 1 mm), and if bleeding or not.

### Blood sampling

In the male study, at 15 weeks of age, cardiac blood samples were collected under inhalant anesthesia into EDTA powdered tubes and the plasma collected and stored at −80 °C until assayed. Plasma samples were shipped on ice to the University of Toronto, Department of Chemistry for PFOS measurement.

### PFOS assay

#### Sample extractions

Mouse plasma samples were extracted using an ion-pair extraction method [17, 18]. Before extraction, mouse plasma samples were diluted with Milli-Q water (i.e., 10-fold for control group and 1000 to 10000-fold for treatment groups). In brief, in a 15 mL polypropylene tube, 1 mL of TBAS solution (adjusted to pH 10 using 30% aqueous NH_3_) was added to 1 mL of the diluted plasma sample; after the mixture was vortex-mixed for 30 s, 5 mL of MTBE was added and was shaken on a horizontal shaker at 250 RPM for 20 min; then the organic and aqueous layers were separated by centrifugation at 6000 RPM for 10 min. The organic layer was decanted to a new tube. The sample was then extracted with another 5 mL aliquot of MTBE and the entire extraction procedure repeated. The MTBE aliquots were combined, evaporated to dryness under a gentle stream of nitrogen, and reconstituted in 1 mL of methanol for analysis.

Drinking water stock solution with 0.5% Tween-20 was extracted using a SPE-WAX cartridge [19]. The cartridge was first conditioned by passing a series of 4 mL of 0.1% NH_4_OH in methanol, 4 mL of methanol, and 4 mL of Milli-Q water; after that, 0.5 mL of the mouse drinking water stock was loaded onto the cartridge. After loading the sample, the cartridge was washed with 4 mL of 25 mM ammonium acetate and dried under vacuum. Target fraction was eluted with 4 mL 0.1% NH_4_OH in methanol and evaporated to dryness, and then reconstituted in 0.1 mL of methanol for analysis.

#### Instrumental analysis

Apart from PFOS, a suite of target Per- and poly-fluorinated alkyl substances, C6-C8 perfluorinated carboxylic acids and perfluorohexane sulfonate were analyzed using an Acquity UPLC (Waters Corporation) and a API 4000 MS/MS (Applied Biosystem/MDS Sciex); an atmospheric electrospray interface operated in negative ionization mode was used. Chromatographic separation was performed on a Kinetex XB-C18 column (50 × 4.6 mm, 2.6 um 100A), the column temperature was kept at 40 °C, and 10 mM ammonium acetate in both Milli-Q water and methanol were the mobile phases.

An internal calibration method using mass-labelled standard was used to quantify PFOS. The calibration curve was constructed with standard concentrations ranging from 0.5, 1.0, 2.5, 5.0, 10.0, 20.0, 50.0, and 100.0 ng/mL. Standard deviations at each data point were < 20%, with an r^2^ > 0.99 for the calibration curve. The PFOS standard used in the present study was the linear isomer, while samples contained both branched and linear isomers; the concentrations reported for the present study included both linear and branched isomers, and were estimated based on the linear isomer standard. The limit of quantification (0.5 ng/mL) was evaluated based on the lowest concentration of standard on the calibration curve that could be accurately measured within ± 20% of its theoretical value and a signal-to-noise ratio ≥ 10.

#### Quality assurance and quality control

Three procedural blanks (Milli-Q water) were run for every twelve sera samples to check for possible interference. Matrix recoveries (*n* = 3) using control mouse sera were performed prior to real sample analysis to ensure the reliability of the method. All target chemicals were spiked (10 ng) into the control samples, and the samples were extracted and analyzed following the same procedures as described above; matrix recoveries ranged 87–117% (supporting information, SI). Samples were analyzed in duplicate and the variability of the analysis was less than 10% as evaluated using ^13^C_4_-PFOS recoveries, which likewise ranged from 88–110%. In response to co-eluting interferences at PFOS transition 499 > 80, the 499 > 99 transition was used for quantification. Recoveries (spiked level: 0.5 ng) for water samples ranged 90–107% (SI) using the same method. A quality control standard (10 ng/mL) was injected every ten samples to evaluate intensity change of the MS; samples were re-analyzed if the intensity of the standard varied ± 20% or more compared to those of the previous one.

### Statistical analyses

Animals from the 2 studies represented different total doses, exposure periods and genders, so each study was analyzed separately. Both datasets were normality tested to assure parametric testing was appropriate. Initial ANOVA analysis was performed looking for treatment effects. Based on these results, summary statistics were calculated (means and standard errors), and assuming unequal variances, pairwise comparisons of treatment groups were made using two-sample T tests corrected using Tukey’s criterion for multiple comparisons. Cecum masses were not included in analyses due to low tumor numbers. Slopes of mean body weights through time from the males exposed to vehicle or 200 mg/kg PFOS from 12–15 weeks were tested to determine if body weight trajectories are significantly different between the two groups.

## Results

**Fig. 1:**
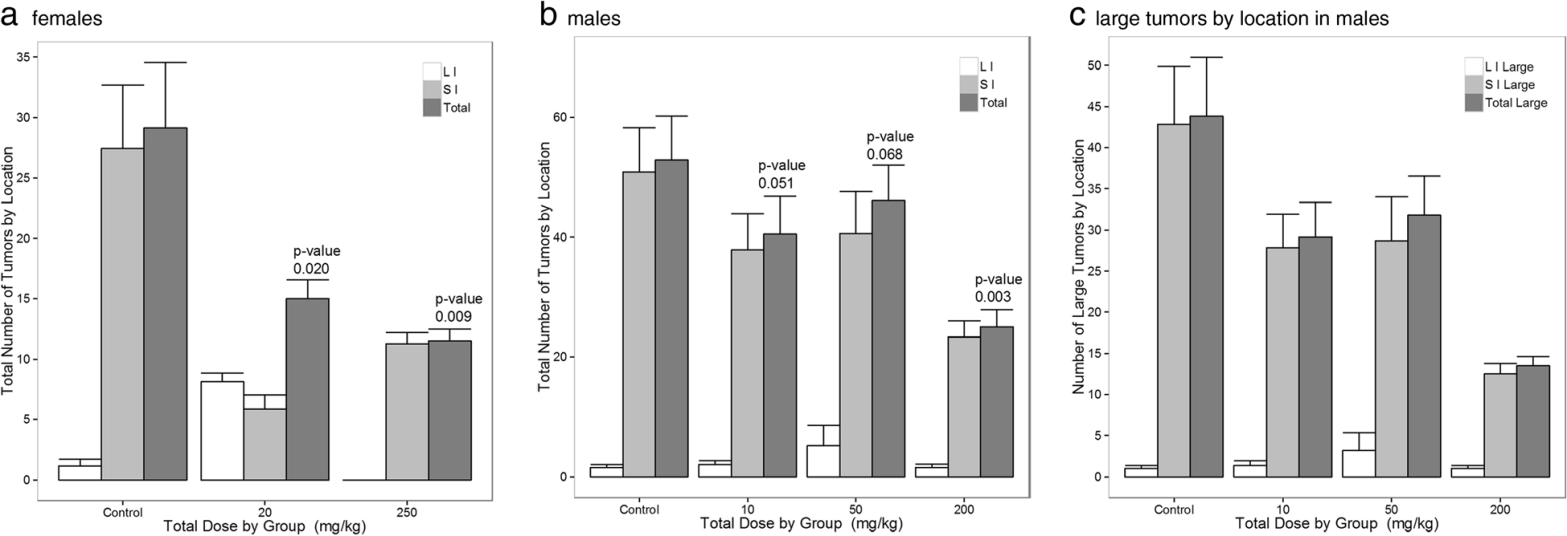
**a-c** Fig. 1a shows the number of tumors (mean and s. e.: Total, SI-small intestine, LI-large intestine) counted at 15 weeks in female APC^min^ mice exposed to varying target doses from 7–15 weeks of age, and receiving up to 250 mg/kg PFOS in their drinking water. Controls received 0.5% Tween-20 vehicle only. Figure 1b shows the number of tumors counted by region (mean and s. e.: Total, SI-small intestine, LI-large intestine) in male APC^min^ mice exposed to target doses of up to 200 mg/kg PFOS in their drinking water. Figure 1c. The number of large tumors 1–3 mm in diameter plotted against PFOS is depicted. These results suggest PFOS may cause tumor regression, and not just prevent tumor development (mean and s. e.: Total, SI-small intestine, LI-large intestine). For the female study, animal numbers were 7, 8, and 4 for vehicle, 20, and 250 mg/kg dose groups respectively. For the male study, animal numbers were *n* = 6 for each group. Values represent total tumors counted in the vehicle controls as compared to each treatment group

Of the original 24 female APC^min^ mice, one from the 20 mg/kg dose group developed breast cancer, and 4 from the 250 mg/kg dose group lost > 10% body weight, so they were lost to follow-up before 15 weeks. Findings reported for this pilot study, illustrated in Fig. 1a, are based on the remaining animals. Initially, ANOVA analysis using treatment as the independent variable and total tumor number as the dependent variable, revealed *p*-values of 0.010 and 0.019 for females and males respectively. At 15 weeks in females, total tumor numbers in both treatment groups averaged significantly lower than those in the control group (*p* = 0.02 and 0.009 for 20 mg/kg and 250 mg/kg, respectively). Likewise, animals in the 250 mg/kg group averaged significantly fewer tumors than in the 20 mg/kg group (*p* = 0.04). Collectively, these findings indicate increasing tumor reductions with rising PFOS dose.

Findings of the study in male mice are shown in Fig. 1b, and suggest a similar dose–response relationship overall.

Again, total tumor load in all treatment groups averaged lower than that in the control group at 15 weeks, with the largest effect in the highest dose group (*p’s* = 0.051, 0.068, and 0.003 for vehicle vs. 10, 50, and 200 mg/kg, respectively). Likewise, while response in the lowest PFOS dose group did not differ significantly from that in the moderate dose group (*p* > 0.1), total tumor count in animals receiving PFOS doses of 200 mg/kg was significantly reduced relative to both the 10 mg/kg (*p* = 0.006) and 50 mg/kg dose groups (*p* = 0.02), again suggesting greater tumor reduction at higher PFOS doses.

As indicated in Fig. 1c, the inverse, dose–response effect of PFOS exposure was particularly pronounced for tumors ≥ 1 mm in size. For males, PFOS showed a strong inverse linear association to large tumor numbers (*r* ^*2*^ = 0.44) with a *p* < 0.001. For tumors < 1 mm, this association did not hold (*p* = 0.76). In both studies, bleeding tumors were rare, appeared to be evenly distributed across dose groups, and their occurrence appeared unrelated to either tumor size or location.

**Fig. 2:**
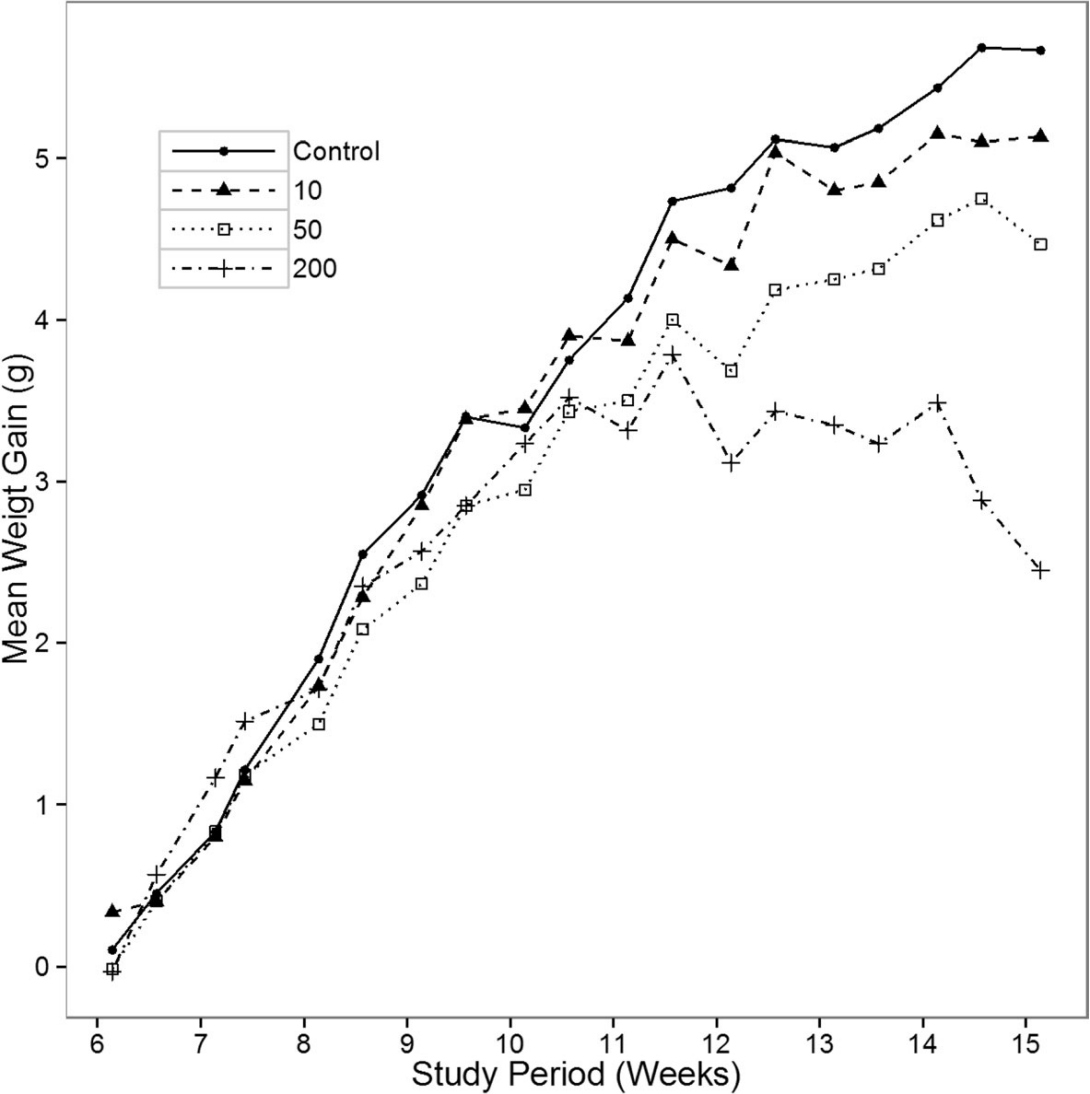
Shown are plasma PFOS levels (mean and s. e.) from male APC^min^ mice at 15 weeks of age by dose administered. Total linear and branched PFOS levels are shown. As expected, levels increased with PFOS dose

**Fig. 3:**
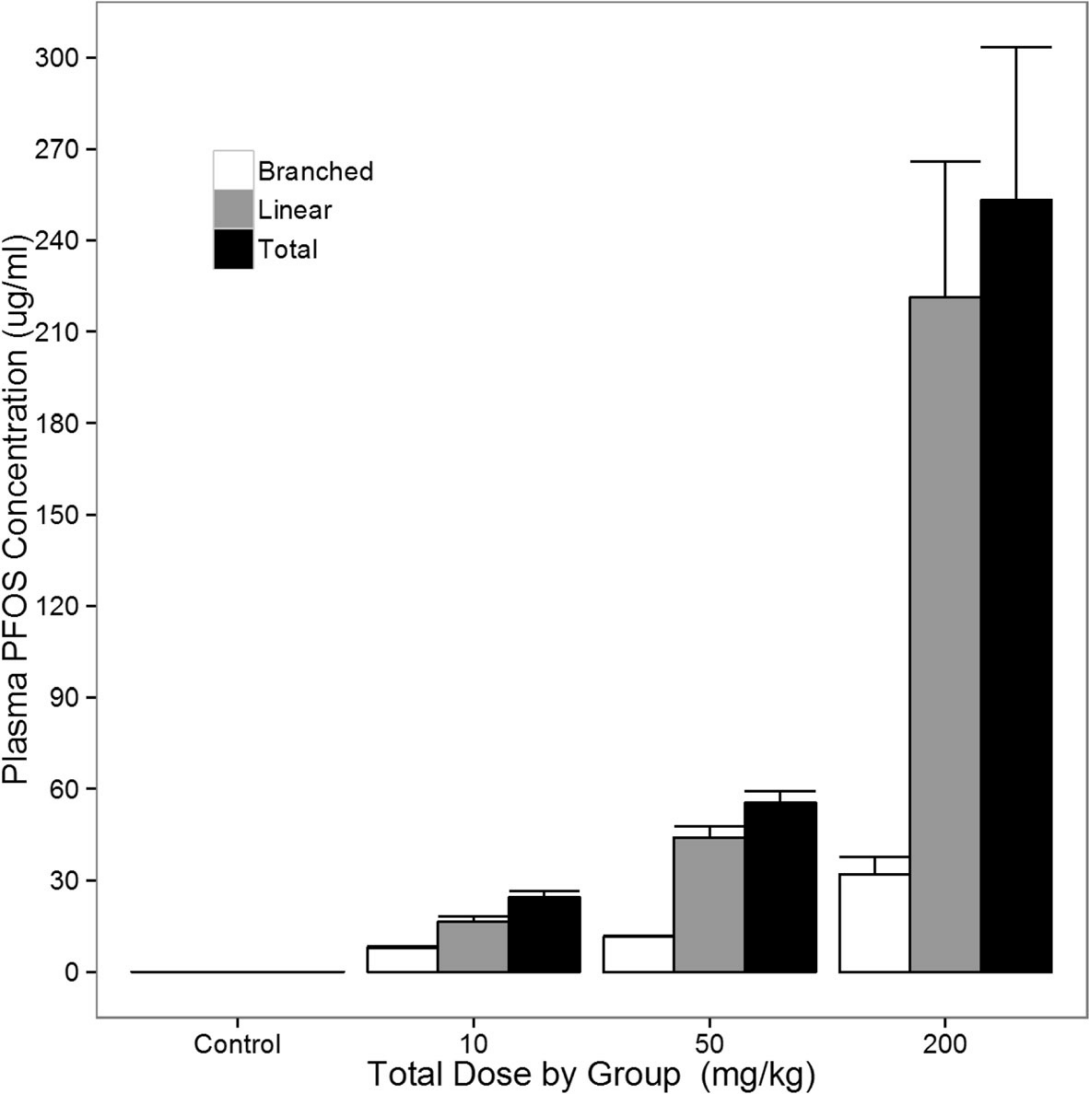
Shown is the average body weight (mean and s. e.) by dose group from 6–15 weeks of age weeks in male mice. As can be seen, body weight appeared to slow in proportion to PFOS dose. At approximately 12 weeks of age, the 200 mg/kg group stopped exhibiting weight increases altogether. Table 1 depicts the gender, dose groups, animal numbers and duration of exposure for the animals completing each study

Figure 2 shows the effect of PFOS dose on body weight in males. Weight gain decreased with dose in the final weeks of the study, with the between group differences increasingly prominent after 11 weeks. As expected, weight gain reductions were particularly pronounced in the 200 mg/kg group, with the slope of body weight change in this group becoming negative by 12 weeks, with body weight trending lower than for the vehicle controls from 12–15 weeks (*p* = 0.06). Weight loss in this high dose group late in the study likely reflects a developing toxic effect of PFOS, and is consistent with the weight loss observed at higher doses in the female study. As illustrated in Fig. 3, plasma PFOS levels at 15 weeks of age indicate that PFOS accumulation increased with dose, including estimated levels of both linear and branched isoforms.

**Table 1:** Depicts the gender, dose groups, animal numbers and duration of exposure for the animals completing each study

Trial	Dose Groups in mg/kg (N)	Age at First Exposure	Treatment Duration	Age at Tumor Counts
Females	0 (8), 20 (7^a^), 250 (4^b^)	7 weeks	8 weeks	15 weeks
Males	0 (6), 10 (6), 50 (6), 200 (6)	6 weeks	9 weeks	15 weeks

^a^One animal developed breast cancer and was dropped from study; ^b^four animals had > 10% weight loss before 15 weeks and were lost to follow-up. “0” dose animals received vehicle only

## Discussion

In these two preliminary studies of male and female APC^min^ mice, total tumor number decreased significantly with increasing PFOS dose, with the highest dose groups showing the largest effects. The observed dose–response relationship was particularly evident in larger tumors, suggesting a possible inhibitory effect of PFOS on tumor formation. Since PFOS administration in our mouse model was initiated prior to tumor development, the observed reduction in tumor burden may reflect effects on both tumor initiation and tumor progression. Notably, the number of 1–3 mm tumors decreased significantly with increasing PFOS dose (Fig. 1c), suggesting that PFOS may not only inhibit development, but may halt progression and even possibly induce tumor regression. If PFOS induces tumor regression, reduction of larger tumors may lead to a corresponding increase in the number of tumors < 1 mm diameter, thus potentially attenuating the observed effect of PFOS on total tumor number and helping to explain the stronger effects observed for larger tumors. Collectively, these findings suggest that PFOS has a significant, dose-dependent inhibitory effect on gastrointestinal tumor formation in this established genetic mouse model of familial adenomatous polyposis.

Results of these preliminary experimental studies are broadly consistent with findings from our recent epidemiological investigation in a large population of Appalachian adults exposed to PFOA-contaminated drinking water. In this cross-sectional study, serum PFOS levels showed a strong, inverse dose–response association with prevalent colorectal cancer that remained robust after adjustment for multiple potential confounders [12]. However, while findings of this epidemiological investigation likewise suggest a possible protective effect of PFOS on colorectal cancer, the cross-sectional nature of the data limit causal inference. Although implications for non-familial colorectal cancer remain unclear, the current study in a mouse model of familial adenomatous polyposis offers the first experimental evidence that chronic exposure to PFOS in drinking water can reduce formation of gastrointestinal tumors, and that these reductions are both significant and dose-dependent.

In our present animal study, we provided PFOS in the drinking water to simulate chronic human PFOS exposure [20, 21]. Liver enzymes can be induced by PFOS exposure in mice [22], and toxicity indicated by weight loss [23] was observed here. In other studies, higher PFOS doses have been administered over shorter periods by oral bolus [5, 24] without evident toxic effects; however, our data suggest toxicity likely develops at a lower overall dose when PFOS is delivered slowly over time [5, 24]. Here progressive weight loss was observed at doses of 200 mg/kg or higher, indicating this dose is near the maximum tolerated dose for this mouse strain and delivery method over this time frame. Fortunately, measurement of plasma PFOS levels in male mice at 15 weeks indicated that drinking water administration at all doses resulted in plasma levels substantially higher than those associated colorectal cancer reduction in humans [12]. In common, PFOS appeared beneficial in human colorectal cancer and in APC^min^ mice. However, the former effect was observed in humans which metabolize PFOS differently from rodents, and where the effect was substantially based on the acquired non-familial form of colorectal cancer. In the mouse model, PFOS undergoes a greater degree of metabolism, has a shorter half-life, and counters a genetic predisposition to colorectal cancer. Further studies should investigate the adverse consequences of this agent under therapeutic conditions; even so, this study provides a possible direction to pursue in regard to familial colorectal cancer.

Although PFOS is widely distributed in the environment [25, 26] and has been detected in human populations worldwide [9, 27–30], non-occupational blood levels in humans are well below those reported toxic in lab animals [31–33]. The half-life of PFOS is reported to be < 40 days in mice [34], and contrasts dramatically with the estimated 4–5 year half-life documented in humans [35]. It appears that PFOS in rodents is handled in a manner similar to fatty acids, and consequently induces hormonal, peroxisomal and P450 enzyme gene activation [36]. The increased PFOS half–life in humans compared to rodents may be in part due to PFOS inhibition of human cytochrome activity [37]; cytochrome activity inhibition could also reduce the influence of toxic metabolites which may explain higher degrees of toxicity as commonly reported in rodents. In addition, PFOS renal reabsorption and recycling have also been shown to contribute to a long half-life in humans and monkeys [38]. Cancer risk with prolonged chronic exposure was suggested at high doses in rats [11], although consistent evidence for elevated tumor risk with PFOS exposure in humans is lacking [11].

Each PFAS has a unique biological and toxicological profile that limits extrapolation across compounds or model species [11]. Potential mechanisms of PFOS action relevant to its effect on tumor development and progression are still ill-defined, but are not surprising given the large number of genes (e.g. ~400 in rats) PFOS appears to influence [36]. Possibilities include anti-inflammatory effects via prostanoid pathways, PPAR receptor mediated actions, immune effects, or other as yet unrecognized mechanisms. PFOS may serve an anti-inflammatory role via its influence on downstream transcriptional regulators such as NF-κB [6]. Phospholipase A2 is inhibited by PFOS in rats; this could, in turn, block the production of arachidonic acid as a substrate for prostaglandindin H synthase elaboration of prostanoids [36]. Similarly, PGE_2_ has been shown to be a potent inducer of adenoma formation in APC^min^ mice [39], and tumor growth [40] was similarly increased by an agonist, where both were mediated through the PPARδ receptor. Adenoma formation by PGE_2_ was removed in mice missing this receptor [41]. PFOS also significantly stimulates both PPARα and PPARγ [42], which could also modulate tumor growth [43, 44]. PFOS serves as a partial agonist and induces PPARα mediated effects at high doses [45], other effects via other PPAR receptor isotypes [46], and produces significant immunomodulatory effects in mice [47]. In PPARα KO mice, PPARα independent nuclear receptor mediated pathways and down-stream effects were noted [6], including suppression of T-cell dependent antibody production, and modulation of immune cell and cytokine synthesis (e.g. TNFα and IL-6) [6]. While PFOS appears to stimulate mouse and human PPAR receptors [42, 46] when screened in cell lines, robust in vivo evidence for direct PPAR receptor mediation is lacking, and human PPARα expression is considerably reduced compared to in rodents; if so, this may thus lessen the importance of this pathway in human familial adenomatous polyposis or acquired colorectal cancer [6]. Even so, PPARα stimulation in human colorectal cancer lines is moderately pro-inflammatory and stimulates prostaglandin H synthase-2 expression [48, 49]. The potential impact of PFOS directly on the Wnt-β-catenin signal transduction pathway also warrants closer examination [50]. The “*min*” defect causes catenin retention and ultimately the Wnt gene group to become canonically activated; gastrointestinal polyp formation is one direct consequence [51]. The putative role of PFOS in blocking this process would be a plausible mechanism to explain its efficacy in this model system. Alternative pathways could also be affected [52]. Other influences of PFOS, by interacting with dietary constituents [53, 54], or steroidogenic enzyme and hormone disruptive effects cannot be ruled in or out [55]. Here, both mouse genders benefited from PFOS exposure, arguing against a differential role as pertains to specific sex hormones.

In recent human cross-sectional studies, chronic PFOS exposure has been associated with modest, adverse changes in serum lipid profiles [56–58]. Similarly, elevated blood levels of PFOS have been associated with increased likelihood of early onset menopause [59] and altered thyroid function [59]. However, in contrast to findings from animal studies, including our study in APC^min^ mice, significant adverse effects of PFOS exposure have been rarely been documented in humans, even in pregnant women and highly exposed fluorochemical plant workers [16, 58, 60, 61]. Even with its complex disposition in humans, PFOS appears well tolerated at environmental levels (e.g. Danish National Birth Cohort Study [62]: PFOS mean 35.3 ng/ml; range 6.4–106.7 ng/ml; elsewhere 0–30 ng/ml [15, 63–65],) levels also associated with colorectal cancer protection [66]. Occupational exposures in chemical factory workers are up to 40 times higher, yet adverse outcomes at these levels, even among at risk pregnant women, are rare [16, 60]. At high occupational exposure levels, an association between PFOS and bladder cancer was reported [65]; however, this association was questioned more recently [16, 58, 61]. Therefore, although PFOS has been decried as an environmental contaminant, it might still have therapeutic value at low levels. If shown to be effective for colorectal cancer prevention and/or treatment in humans, PFOS may offer an option that is significantly safer, lower cost, and less toxic than alternative therapies [67].

One potential limitation relates to reverse causality, i.e., the possibility that tumor formation may reduce PFOS absorption, and thus, blood PFOS levels. However, this is unlikely given the massive surface area of the gastrointestinal tract and the inherent lipid solubility of PFOS. Moreover, serum PFOS generally correlates well with liver concentrations [61], suggesting that serum is a reasonably good systemic indicator of PFOS exposure in humans [68]. Similarly in a mouse model of familial adenomatous polyposis, blood levels corresponded to target dosing levels and predicted tumor reduction, again indicating that PFOS absorption reflects oral exposure. Another theoretical argument is that a parent compound is broken down to make PFOS, and it may be this compound, rather than PFOS itself that led to the reduction in colorectal cancer observed in our previous epidemiological study [12]. However, PFOS exposure had a clear benefit here.

Strengths of this study include identifying a beneficial role for PFOS as a chemo-preventive or therapy in a familial model of colorectal cancer, irrespective of gender. In addition, the development of a slow delivery method and estimates of an effective working dose range by this delivery method were determined. Finally, toxic effects were easily identified using simple body weight monitoring. Limitations include the absence of more dose groups to better define the dose–response relationship, with the eventual goal of developing a reliable PFOS therapeutic profile.

While effective against this animal model of familial adenomatous polyposis, PFOS efficacy in the more common acquired form of colorectal cancer is another important focus for future investigations. Planned studies will seek to determine a mechanism, such as might be derived from isolated cell cultures, an optimal dose in vivo using xenograft models. A mechanistic understanding of PFOS action, from the impetus provided here, may lead to successful new colorectal cancer treatment approaches.

## Conclusions

Using APC^min^ mice of both genders, we show that perfluorooctane sulfonate (PFOS) reduces gastrointestinal tumor burden in this well-established general model of human colorectal cancer, and also a specific model for familial adenomatous polyposis in a dose-related manner. Our results represent a proof of concept based on our previously published epidemiological study that found a protective dose–response relationship between PFOS levels and reduced likelihood of colorectal cancer from a human population.

## Abbreviations

ANOVA: Analysis of variance
C57BL/6 J-Apc^Min^/J mice: APC^min^ mice
LCMS: Liquid chromatography mass spectrometry
MTBE: Methyl-tert-butyl ether
*PFOA*: Perfluorooctanoic acid
PFOS, C_8_HF_17_O_3_S: Perfluorooctane sulfonate
PPAR: Peroxisome proliferator-activated receptor
SPE: Solid phase extraction
WAX: Oasis® weak anion exchange
